# Do the patient education program and nurse-led telephone follow-up improve treatment adherence in hemodialysis patients? A randomized controlled trial

**DOI:** 10.1186/s12882-021-02319-9

**Published:** 2021-04-07

**Authors:** Mansour Arad, Rasoul Goli, Naser Parizad, Davoud Vahabzadeh, Rahim Baghaei

**Affiliations:** 1 Department of Medical-Surgical Nursing, School of Nursing and Midwifery, Urmia University of Medical Sciences, Urmia, Iran; 2 Patient Safety Research Center, Clinical Research Institute, Nursing & Midwifery School, Urmia University of Medical Sciences, Urmia, Iran; 3 Nutrition & Biochemistry Department, School of Medicine, Ilam University of Medical Sciences, Ilam, Iran

**Keywords:** Tele-nursing, Education, Chronic kidney disease, Hemodialysis, Patient, Trial

## Abstract

**Background:**

End-Stage Renal Disease (ESRD) is the final and permanent stage of Chronic Kidney Disease (CKD). Hemodialysis (HD) is the most common treatment for CKD. To have desirable therapeutic outcomes, patients have to adhere to a specific therapeutic regimen that reduces the hospitalization rate and side-effects of HD. The present study aimed to determine the effects of the patient education program and nurse-led telephone follow-up on adherence to the treatment in hemodialysis patients.

**Methods:**

This is a randomized controlled trial in which a total of 66 patients were recruited using convenience sampling and then randomly assigned to two groups of control (*n* = 33) and intervention (*n* = 33). Data were collected using a demographic questionnaire, the laboratory results record sheet, and the End-Stage Renal Disease Adherence Questionnaire (ESRD-AQ), which included four dimensions of HD attendance, medication use, fluid restrictions, and diet recommendations. The intervention group received a patient education program and nurse-led follow-up services through telephone communication and the Short Message Service (SMS) for 3 months. All participants filled in the questionnaire before and after the intervention. Data were analyzed using IBM SPSS Statistics for Windows, version 25 (IBM Corp., Armonk, N.Y., USA).

**Results:**

The results showed a significant difference in the mean scores of HD attendance, medication use, fluid restrictions, and diet recommendations between the two groups immediately, 1 month, and 3 months after the intervention (*p* < .001). The results also indicated a significant difference in the mean scores of four dimensions during the four-time points of measurement in the intervention group (*P* < 0.0005). Therefore, the level of treatment adherence in the intervention group was higher than in the control group. Moreover, there was a significant difference in the mean score of laboratory values between the two groups after the intervention, except for the level of serum sodium (*P* = 0.130).

**Conclusion:**

Implementation of the patient education program and nurse-led follow-up can lead to better adherence to hemodialysis in four dimensions of HD attendance, medication use, fluid restrictions, and dietary recommendations in HD patients.

**Trial registration:**

IRCT registration number: IRCT20190127042512N1; Registration date: 2020-09-12; Registration timing: retrospectively registered: Last update: 2020-09-12.

**Supplementary Information:**

The online version contains supplementary material available at 10.1186/s12882-021-02319-9.

## Highlights

- Dialysis patients usually do not adhere to their therapeutic regimen.
- The nurse’s educational program is not sufficient.
- Telephone follow-up can improve patients’ adherence to the dialysis treatment.

## Background

Chronic Kidney Disease (CKD) is a general health risk factor worldwide with a higher prevalence in individuals older than 60 years old [1, 2]. The CKD refers to a type of kidney disease in which the kidneys lose more than 50% of normal function [3] and is defined as the presence of kidney damage or an estimated Glomerular Filtration Rate (eGFR) below 60 ml/min/1.73 m^2^ persisting at least for 3 months [4].

Patients with CKD experience different stages of the disease (one to five), which is figured out based on the eGFR level [5]. Stages 3 to 5 of CKD have considerable negative effects on patients’ activities of daily living, health status, nutrition, and water and electrolyte hemostasis, which can cause uremic syndrome (uremia) and result in death if not treated [6]. End-Stage Renal Disease (ESRD) is the final stage of CKD and is defined as an irreversible decrease in kidney function as there is a need for a regular course of long-term hemodialysis or a kidney transplant to sustain life [7].

The ESRD is one of the major public health problems worldwide, and it can cause considerable financial stress for the societies and health systems [8]. Based on the National Health and Morbidity Survey reports, the prevalence of CKD has increased from 9.1% in 2011 to 15.5% in 2018. The incidence and prevalence of ESRD have also increased notably over the last 25 years and the number of ESRD patients is estimated to reach 51,000 in 2020 and 106,000 in 2040 [9]. In Iran, the prevalence and incidence rate of ESRD is about 357 and 57 per million each year, respectively [10]. According to the health statistics, the number of CKD patients in Iran was more than 55,000 in 2016, out of which 27,500 received Hemodialysis (HD) and 1600 received Peritoneal Dialysis (PD) [11].

Further studies have shown considerable growth in the number of CKD patients in Iran, as the number of these patients increases by 15% every year [12]. Currently, HD is the most common method of treatment for ESRD patients [13]. However, patients receiving HD have to deal with several issues and changes in their lives. To avoid cardiovascular complications caused by HD, the patients need to adhere to a special diet [14].

Establishing a successful HD program depends on adherence to four factors of diet, medication use, fluid restrictions, and HD attendance [15]. Adherence to the therapeutic regimen in ESRD patients is a major factor in achieving preferred therapeutic outcomes. It decreases the hospitalization rate, debilitation, and side-effects such as nutritional disorders, muscle spasm, and blood infection [16].

Failure to adhere to the therapeutic regimen is a major problem in chronic patients, including HD ones. More than half of the HD patients fail to observe their therapeutic regimen [17]. Different factors affect HD patients ‘adherence to the therapeutic regimen. These factors include knowledge about the therapeutic regimen, socioeconomic status, health beliefs, attitude towards treatment, and culture. Adherence to dietary recommendations, fluid restrictions, and medication is not easy for patients, and failure to do so can cause great risks [18].

Regarding the patients’ limited health knowledge, they have little control over the disease and its complications. Therefore, patient education can lead to a higher level of satisfaction, a better quality of life, assurance of the care continuity, reduced anxiety, less severe complications, attendance healthcare programs, client independence in doing daily activities, better provision of healthcare, and a decrease in treatment costs [19].

In Iran, the provision of educational courses about therapeutic regimens for CKD patients is not well-performed by medical professionals because of a large number of patients, lack of time, and ward overcrowding [6]. Therefore, quality patient education requires proper health education methods to ensure a patient-centered interaction and fulfillment of patients’ educational needs [20].

Tele-nursing is one of the methods that rely on information technology. Understaffed wards, increased prevalence of chronic diseases, and population aging in the world entail a proper management to cut medication costs. Long-distance from health facilities and changes in health policies have soared the popularity of healthcare at home and tele-nursing. Healthcare can be shifted from hospital-centered to community-centered care and from care-centered to patient-centered care model through information technology, [21, 22]. Tele-nursing relies on numerous communication tools such as radio, TV, computer, smartphone, and telephone. The key factor in successfully monitoring the patients remotely is to apply an easy-to-use tool by the user. In addition, there should be no need for intensive training on how to use the tool [23]. Tele-nursing refers to applying telecommunication technology in nursing to deliver and improve care services to the patients. The nurse-led telephone follow-up is a well-known care intervention in tele-nursing, as this technology (telephone) is now widely available [24].

Patient education and follow-up care plays a key role in rehabilitation after hospital discharge. Over the past few years, several studies have been conducted in Iran on tele-nursing that showed patient education through booklets was not enough to improve treatment adherence in the patients, and there is a crucial need to implement follow-up methods after hospital discharge [25].

Regarding the importance of adherence to the therapeutic regimen and the role of patient education and nurse-led follow-up intervention in patients undergoing HD, the present study was conducted to determine the effects of the patient education program and nurse-led telephone follow-up on adherence to the treatment in hemodialysis patients.

The alternative hypothesis states that the mean score of treatment adherence in the intervention group differs significantly after conducting patient education program and nurse-led telephone follow-up.

## Methods

### Study design and setting

This is a single-blinded, randomized controlled trial conducted from April 2019 to May 2020 in Taleghani Hospital in Urmia, Iran.

### Participants

In the present study, the target population consisted of HD patients admitted to the dialysis ward of the hospital. Considering the confidence interval of 95% and the power of 80% in the study by Zamanzadeh et al. (2017), the minimum sample size was calculated to be 56 using G*Power 3.1 (Erdfelder, Faul, & Buchner, 1996). Regarding the attrition rate of 20%, the final sample size was considered to be 66 (*n* = 33 per group) [26]. Inclusion criteria were composed of the followings: (a) willingness to participate in the study, (b) being literate, (c) being enough conscious and oriented to answer the questions, (d) having no history of hearing and vision impairments, (e) having no cognitive disorder, (f) having a personal mobile phone and the ability to use it, (g) using no psychedelic drugs, (h) exact diagnosis of CKD confirmed by a nephrologist and having a medical record in dialysis ward, (i) get hemodialysis three times a week in sessions of 3 to 4 h, and (j) being in the 18–65 age group. Exclusion criteria consisted of (a) withdrawal from the study at any phase, (b) failure to receive two consecutive messages/calls, (c) patient death, and (d) being transferred to another health facility.

### Data collection

Data were collected using a demographic questionnaire, the End-Stage Renal Disease Adherence Questionnaire (ESRD-AQ), and the laboratory results record sheet.

The demographic questionnaire consisted of items on age, gender, marital status, education, residency, occupation, dialysis vintage, and the burden of comorbidities.

The ESRD-AQ is a self-report tool that consisted of 46 items in four sections and was designed to evaluate treatment adherence in four dimensions of HD attendance, medication use, fluid restrictions, and diet recommendations (see Additional file 1). The first section seeks general information on patients’ ESRD and history of renal replacement therapy (5 items), and the remaining four sections inquire about treatment adherence in four dimensions of HD attendance (14 items), medication use (9 items), fluid restrictions (10 items), and diet recommendations (8 items). Responses to this tool are based on a combination of Likert scale, multiple-choice, and “yes/no” answer format. The overall score ranges from 0 to 1200, and a higher score indicates higher levels of treatment adherence [27]. Rafiee et al. (2014) confirmed the reliability of the tool using Cronbach’s alpha coefficient (α = 0.91). Moreover, the test-retest reliability coefficient was calculated to be 0.85 [28]. Kim et al. (2010) also examined the content validity of the ESRD-AQ by calculating the Content Validity Index (CVI = 0.99) [27].

The laboratory results record sheet includes laboratory values of serum sodium, potassium, calcium, creatinine, phosphate, albumin, iron, Blood Urea Nitrogen (BUN), hemoglobin, the normalized protein catabolic rate (nPCR), and Kt/V (K: dialyzer clearance, t: dialysis time, V: distribution volume of urea).

### Intervention

After obtaining approval from the Ethics Committee and Vice-Chancellor for Research of Urmia University of Medical science, the researcher referred to the hospital and obtained permission from the hospital officials. The researcher then gave clear explanations of the study process to the head nurse of the dialysis ward. In this phase, convenience sampling was utilized to recruit the patients. Then, eligible patients were invited to participate in the study. The researcher provided clear explanations of the study methodology and objective for the participants and answered their concerns and questions. All participants were also assured of confidentiality and anonymity of all personal information received. Next, the written informed consent was obtained from all participants or their legally acceptable representatives. Subsequently, the patients were allocated to two groups of control (*n* = 33) and intervention (*n* = 33) using sealed envelope randomization. In this randomization method, the researcher used two cards (A, B) to assign the patients to two intervention and control groups randomly. The randomization was performed by having the patient pick one of the two cards inside opaque sealed envelopes. Patients who picked card (A) entered the intervention group, and those who picked card (B) entered the control group.

To prevent contact between the two groups’ patients, the sampling was conducted based on the HD schedules, as the patients of the control and the intervention group were sampled on odd and even days, respectively.

Before the intervention, the laboratory values were collected from patients’ medical records, and all participants filled in the demographic questionnaire and the ESRD-AQ. Data collection for each participant lasted about 30 min.

**Fig. 1:**
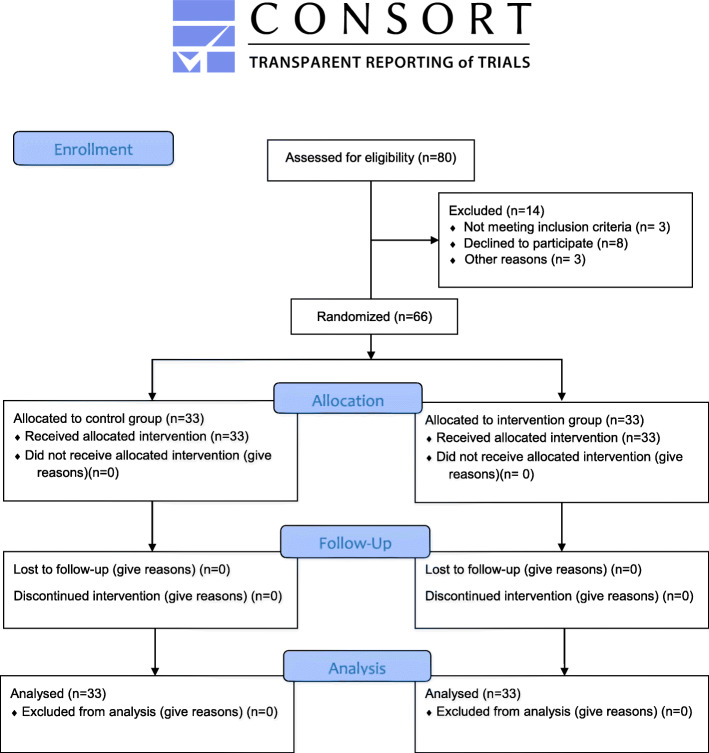
Research flow diagram based on Consort statement 2010

The researcher continued recruitment and randomization until reaching the target sample size (*n* = 66). Sampling was conducted from 9 April to 27 November 2019. Among 80 eligible patients, eight patients declined to participate in the study, three patients did not meet the inclusion criteria, and three patients were transferred to another health facility due to underlying health conditions. *Flow diagram of entering subjects in the study groups has shown in Fig.* *1*.

Patients in the intervention group received the patient education program on the diet, medication use, and fluid restrictions using a patient education booklet. They were also asked to provide their contact information i.e., phone number (mobile/landline number). They were then requested to change their phone language to Farsi and informed of how to use the Short Message Service (SMS). A test text message was sent to the participants’ mobile phones to ensure that the text messages are delivered to them. The message delivery report was also turned on for all participants. In addition, they were recommended to contact the researcher in the case of any question or problem.

Mobile phone and landline telephone were used to perform tele-nursing and the follow-up program. The intervention lasted for 3 months. The researcher contacted the participants twice a week using the telephone. Besides, the researcher telephoned the participants at their convenience, and each phone call lasted for approximately 20 min. In the case of any problem, the participants were provided with effective solutions by the researcher. The text messages concerned patient education topics on diet, medication use, and fluid restrictions. The participants received a text message every day, so that a total of 90 text messages were sent to them during a period of 3 months.

The maximum word number of each message did not exceed 160 characters and the message was marked when it was delivered to the patients. In the case that more than two messages were not delivered, the participant would be contacted using the landline number to check and receive a new mobile number. If the new mobile number also had a problem with receiving text messages, the participant would be excluded from the study. Participants in the control group only received routine care. Routine care in the dialysis unit includes dialysis treatment and answering patients’ questions during and after the treatment. We have no other educational intervention in the dialysis unit. All the participants completed the ESRD-AQ again immediately, 1 month, and 3 months after the intervention in the hospital. We prepared the same educational contents including the diet, medication use, and fluid restrictions, as a self-study booklet and handed it to the participants in the control group after intervention finished and data were collected. The laboratory values were examined and collected again from the medical records. The CONSORT 2010 checklist was used to ensure quality reporting in the present study [29] (see Additional file 2).

### Data analysis

All data obtained from 66 participants were entered into the analysis. The Kolmogorov–Smirnov test was used to examine the normality of data distribution. A researcher, who was blinded to the data, conducted the analysis. All data were entered into IBM SPSS Statistics for Windows, version 25.0 (IBM Corp., Armonk, N.Y., USA). Data were analyzed using descriptive and inferential statistics. In descriptive statistics, we used the frequency and percentage for analyzing qualitative variables and the mean and standard deviation for analyzing normal quantitative variables. We used the Kolmogorov–Smirnov test and confirmed the normal distribution of data. In inferential statistics, we utilized the chi-squared (χ2) test and Fisher’s exact test to assess the homogeneity of the groups. The independent-samples t-test was used to compare the data between the two groups. We also used the paired-samples t-test to compare the mean score within the groups. We used repeated measures ANOVA for studying the changes in the mean score of the treatment adherence during the four-time points of measurement. Analysis of covariance (ANCOVA) was used to adjust confounding variables.

## Results

### Demographic characteristics

The results showed that the overall mean age of the participants in the control and intervention groups were 30 ± 9/5 and 27 ± 11/5 years, respectively. Moreover, in the control group (57.6%), as in the intervention group (54.5%), the majority of participants were male, and also in the control and intervention groups, 87.9 and 97% of the participants were married, respectively. In addition, in both groups, 69.7% of the participants lived in the city. In terms of education level, more than half of the participants (54.5%) had a high school diploma and lower in the control group, but only 39.4% had a high school diploma and lower in the intervention group. In addition, in both groups, the most dialysis vintage was related to 2–5 years, and in the control and intervention groups, the highest comorbidity was related to hypertension (21.2%) and diabetes (21.2%), respectively (Table 1).

**Table 1:** Comparison of demographic characteristics of the patients in the study groups

Variables	Groups		Sig.
	Control	Intervention	
Age	Mean ± SD	Mean ± SD	*P-value = 0.038
	30 ± 9/5	27 ± 11/5	
	n (%)	n (%)	
Gender			
Male	19 (57.6)	18 (54.4)	**P-value = 0.804
Female	14 (42.4)	15 (45.5)	
Education level			
High school Diploma and lower	18 (54.5)	13 (39.4)	**P-value = 0.290
Associate Degree	10 (30.3)	10 (30.3)	
Bachelor’s Degree and upper	5 (15.2)	10 (30.3)	
Marital status			
Single	4 (12.1)	1 (3.0)	***P-value = 0.355
Married	29 (87.9)	32 (97.0)	
Occupation			
Unemployed/ temporary worker	10 (30.3)	10 (30.3)	**P-value = 0.668
Public employee	2 (6.1)	3 (9.1)	
Freelancer	10 (30.3)	6 (18.2)	
Retired	11 (33.3)	14 (42.4)	
Residential status			
Urban	23 (69.7)	23 (69.7)	**P-value = 1.00
Rural	10 (30.3)	10 (30.3)	
Dialysis vintage			
0–6 month	2 (6.1)	3 (9.1)	***P-value = 0.752
6–24 month	8 (24.3)	7 (21.2)	
2–5 year	13 (39.3)	12 (36.4)	
> 5 year	10 (30.3)	11 (33.3)	
Comorbidity			
Hypertension	7 (21.2)	6 (18.1)	**P-value = 0.463
Ischemic heart disease	2 (6.1)	2(6.1)	
Diabetes	6 (18.1)	7 (21.2)	
Cerebrovascular disease	5 (15.1)	4 (12.1)	
Respiratory condition	4 (12.1)	4 (12.1)	
Heart failure	3 (9.1)	2 (6.1)	
Depression	1 (3.1)	2 (6.1)	
Anemia	4 (12.1)	4 (12.1)	
Other	1 (3.1)	2 (6.1)	

* Independent sample t-test

** Chi-squared test

***Fisher’s exact test

The results also indicated that there was no statistically significant difference between the two groups in terms of gender, marital status, education, residency, and occupation (*p > .05*). However, in terms of age, the difference was statistically significant between the two groups (*p = .038*) (Table 1).

Based on ANCOVA results, after adjusting the effect of age, education level, occupational status, and the dialysis vintage, the difference between treatment adherence and its dimensions score was significant between the two groups. Thus, the increase in the mean score of treatment adherence was due to the intervention’s effect.

### Treatment adherence to HD

The results of the independent-sample t-test showed that there was no statistically significant difference in the mean score of HD attendance between the two groups before the intervention (*P* = 0.269). However, the difference was statistically significant immediately, 1 month, and 3 months after the intervention (*P* < 0.001). The results of repeated measures ANOVA also indicated a significant difference in the mean score of HD attendance during the four-time points of measurement in the intervention group (*P* < 0.0005) compared to the control group (*P* = 0.113). Despite a slight reduction in the mean score of HD attendance before the intervention and immediately after the intervention, the Bonferroni post-hoc test indicated that the difference was not significant (*p* = 0.671) (Table 2).

**Table 2:** Comparison of the mean score of the treatment adherence to HD in the study groups

Time of measurement	Groups		Sig.
	Control (*n* = 33)	Intervention (*n* = 33)	
	Mean ± SD	Mean ± SD	
Before intervention	236.36 ± 76.58	257.84 ± 94.69	*P-value =0.269
After intervention	323.48 ± 77.54	468.78 ± 70.26	**P-value < 0.001
One month after intervention	343.93 ± 70.71	531.81 ± 61.64	**P-value < 0.001
Three months after intervention	350.00 ± 69.87	556.06 ± 55.56	**P-value < 0.001
Sig.	***P-value =0.113^1^	***P-value < 0.0005^2^	

* Independent sample t-test

** ANCOVA test

*** Repeated measures ANOVA

^1^ Mauchly’s Test of Sphericity showed that the hypothesis of sphericity had not been rejected

^2^ Mauchly’s Test of Sphericity showed that the hypothesis of sphericity had been rejected. Hence, a Greenhouse-Geisser correction was used

### Medication adherence

Based on the results of the independent-sample t-test, the mean score of medication adherence was not significantly different between the two groups before the intervention (*P* = 0.466). However, this difference was statistically significant immediately, 1 month, and 3 months after the intervention (*P* < 0.001). The results of repeated measures ANOVA also indicated a significant difference in medication adherence during the four-time points of measurement in the intervention group (*P* < 0.0005) compared to the control group (*P* = 0.132). Despite a slight decrease in the mean score of medication adherence before the intervention and immediately after the intervention, the Bonferroni post-hoc test indicated that the difference was not significant (*p* = 0.541) (Table 3).

**Table 3:** Comparison of the mean score of the medication adherence in the study groups

Time of measurement	Groups		Sig.
	Control (*n* = 33)	Intervention (*n* = 33)	
	Mean ± SD	Mean ± SD	
Before intervention	75.75 ± 33.35	83.33 ± 38.86	*P-value =0.466
After intervention	84.84 ± 34.19	159.09 ± 34.12	**P-value < 0.001
One month after intervention	89.39 ± 32.49	187.87 ± 21.75	**P-value < 0.001
Three months after intervention	96.90 ± 27.78	192.42 ± 18.20	**P-value < 0.001
Sig.	***P-value =0.132^1^	***P-value < 0.0005^2^	

* Independent sample t-test

** ANCOVA test

*** Repeated measures ANOVA

^1^ Mauchly’s Test of Sphericity showed that the hypothesis of sphericity had not been rejected

^2^ Mauchly’s Test of Sphericity showed that the hypothesis of sphericity had been rejected. Hence, a Greenhouse-Geisser correction was used

### Fluid restrictions

The independent-sample t-test results also showed that the mean scores of the adherence to fluid restrictions were not significantly different between the two groups before the intervention (*P* = 0.247). However, the difference was statistically significant immediately, 1 month, and 3 months after the intervention (*P* < 0.001). Based on the results of repeated measures ANOVA, there was a significant difference in adherence to fluid restrictions during the four-time points of measurement in the intervention group (*P* < 0.0005) compared to the control group (*P* = 0.126). Despite a slight reduction in the mean score of adherence to fluid restrictions before the intervention and the immediately after the intervention, the Bonferroni post-hoc test revealed that the difference was not significant (*p* = 0.643) (Table 4).

**Table 4:** Comparison of the mean score of the fluid restrictions in the study groups

Time of measurement	Groups		Sig.
	Control (*n* = 33)	Intervention (*n* = 33)	
	Mean ± SD	Mean ± SD	
Before intervention	89.39 ± 36.99	100.00 ± 37.50	*P-value =0.247
After intervention	92.42 ± 35.62	121.21 ± 43.35	**P-value < 0.001
One month after intervention	95.45 ± 31.53	127.27 ± 45.22	**P-value < 0.001
Three months after intervention	98.48 ± 34.19	140.90 ± 40.41	**P-value < 0.001
Sig.	***P-value =0.126^1^	***P-value < 0.0005^1^	

* Independent sample t-test

** ANCOVA test

*** Repeated measures ANOVA

^1^Mauchly’s Test of Sphericity showed that the hypothesis of sphericity had not been rejected

### Diet recommendations

The results of the independent-sample t-test revealed that the mean scores of the adherence to diet recommendations were not significantly different between the two groups before the intervention (*P* = 0.136). However, in this term, a significant difference was found immediately, 1 month, and 3 months after the intervention (*P* < 0.001). In terms of adherence to diet recommendations, the results of repeated measures ANOVA indicated a significant difference during the four-time points of measurement in the intervention group (*P* < 0.0005) compared to the control group (*P* = 0.344). Despite a slight decrease in the mean score of the adherence to diet recommendations before the intervention and immediately after the intervention, the Bonferroni post-hoc test showed that the difference was not significant (*p* = 0.431) (Table 5).

**Table 5:** Comparison of the mean score of the diet recommendations in the study groups

Time of measurement	Groups		Sig.
	Control (*n* = 33)	Intervention (*n* = 33)	
	Mean ± SD	Mean ± SD	
Before intervention	112.12 ± 43.35	125.75 ± 28.24	*P-value =0.136
After intervention	115.15 ± 36.41	154.54 ± 36.14	**P-value < 0.001
One month after intervention	128.78 ± 35.42	163.63 ± 36.95	**P-value < 0.001
Three months after intervention	134.84 ± 31.83	168.19 ± 30.15	**P-value < 0.001
Sig.	***P-value = 0.344^1^	***P-value < 0.0005^2^	

* Independent sample t-test

** ANCOVA test

*** Repeated measures ANOVA

^1^Mauchly’s Test of Sphericity showed that the hypothesis of sphericity had not been rejected

^2^Mauchly’s Test of Sphericity showed that the hypothesis of sphericity had been rejected. Hence, a Greenhouse-Geisser correction was used

### *Total* treatment *adherence*

Eventually, the results of the independent-sample t-test showed that there was no statistically significant difference in the mean score of overall treatment adherence between the two groups before the intervention (*P* = 0.436). However, the difference showed to be statistically significant immediately, 1 month, and 3 months after the intervention (*P* < 0.001). In this regard, repeated measures ANOVA results indicated that there was a significant difference in the mean score of overall treatment adherence during the four-time points of measurement in the intervention group (*P* < 0.0005) compared to the control group (*P* = 0.076) (Table 6).

**Table 6:** Comparison of the mean score of the total treatment adherence in the study groups

Time of measurement	Groups		Sig.
	Control (*n* = 33)	Intervention (*n* = 33)	
	Mean ± SD	Mean ± SD	
Before intervention	513.63 ± 125.52	566.93 ± 117.43	*P-value =0.436
After intervention	615.90 ± 113.50	903.63 ± 91.89	**P-value < 0.001
One month after intervention	650.57 ± 31.53	1010.60 ± 82.68	**P-value < 0.001
Three months after intervention	680.30 ± 107.43	1057.57 ± 80.64	**P-value < 0.001
Sig.	***P-value =0.076^1^	***P-value < 0.0005^2^	

* Independent sample t-test

** ANCOVA test

*** Repeated measures ANOVA

^1^ Mauchly’s Test of Sphericity showed that the hypothesis of sphericity had not been rejected

^2^Mauchly’s Test of Sphericity showed that the hypothesis of sphericity had been rejected. Hence, a Greenhouse-Geisser correction was used

### Laboratory values

Based on the results of the independent-samples t-test, there was a significant difference in the mean score of laboratory values between the two groups after the intervention, except for the level of serum sodium (*P* = 0.130). The paired-samples t-test also showed a significant difference in all laboratory values before and after the intervention in the intervention group, although in the control group, the only significant difference was found in the mean score of serum calcium (*P* = 0.005) and iron (*P* < 0.001) (Table 7). In the present study, no ESRD side-effects were reported.

**Table 7:** Comparison of the mean score of laboratory values in the study groups before and after the intervention

Variables	Time of measure-ment	Groups		Sig.
		Control (*n* = 33)	Intervention (*n* = 33)	
		Mean ± SD	Mean ± SD	
Serum Sodium (mEq/L)	Before	150.84 ± 4.55	151.42 ± 5.43	*P-value =0.642
	After	145.96 ± 14.78	141.84 ± 3.76	*P-value = 0.130
Sig.		**P-value = 0.087	**P-value< 0.001	–
Serum Potassium (mEq/L)	Before	5.07 ± 0.57	4.97 ± 0.56	*P-value =0.462
	After	5.50 ± 0.41	4.49 ± 0.47	*P-value < 0.001
Sig.		**P-value = 0.952	**P-value = 0.001	–
Serum Calcium (mg/dL)	Before	7.10 ± 0.66	7.28 ± 0.87	*P-value =0.215
	After	7.99 ± 1.67	8.97 ± 0.68	*P-value =0 .003
Sig.		**P-value = 0.005	**P-value = 0.001	–
Serum Creatinine (mg/dL)	Before	3.43 ± 1.65	3.00 ± 1.19	*P-value =0.240
	After	2.91 ± 0.68	1.86 ± 0.41	*P-value < 0.001
Sig.		**P-value = 0.063	**P-value< 0.001	–
Serum Iron (μg/dL)	Before	54.35 ± 8.26	55.83 ± 6.61	*P-value =0.426
	After	65.33 ± 10.41	118.71 ± 17.44	*P-value < 0.001
Sig.		**P-value< 0.001	**P-value< 0.001	–
BUN (mg/dL)	Before	49.28 ± 7.58	50.36 ± 8.64	*P-value =0.080
	After	48.41 ± 11.51	37.98 ± 11.19	* *P*-value < 0.001
Sig.		**P-value = 0.726	**P-value< 0.001	–
Serum Phosphate (mg/dL)	Before	6.23 ± 1.65	6.32 ± 1.58	*P-value =0.821
	After	6.46 ± 1.55	5.49 ± 1.32	* *P*-value < 0.001
Sig.		**P-value = 0.863	**P-value< 0.001	–
Hemoglobin (g/dL)	Before	10.36 ± 1.5	10.40 ± 1.6	*P-value =0.324
	After	10.34 ± 1.7	11.04 ± 1.3	* *P*-value < 0.001
Sig.		**P-value = 0.901	**P-value< 0.001	–
Serum Albumin (g/dL)	Before	4.85 ± 0.65	4.72 ± 0.86	*P-value =0.541
	After	4.33 ± 0.58	5.11 ± 0.41	* *P*-value < 0.001
Sig.		**P-value = 0.621	**P-value< 0.001	–
nPCR (g/kg/d)	Before	1.77 ± 0.65	1.79 ± 0.61	*P-value =0.931
	After	1.78 ± 0.59	1.14 ± 0.32	*P-value =0.011
Sig.		**P-value = 0.843	**P-value = 0.041	–
Kt/V	Before	1.34 ± 0.25	1.31 ± 0.28	*P-value =0.788
	After	1.35 ± 0.26	1.84 ± 0.27	* *P*-value < 0.001
Sig.		**P-value = 0.776	**P-value = 0.001	–

* Independent sample t-test

** Paired sample t-test

## Discussion

Tele-nursing is becoming a new method for providing nursing care, and it has been increasingly used as an effective approach to chronic disease care. The results of this study indicated that the patient education program and nurse-led telephone follow-up could improve treatment adherence in the four dimensions of HD attendance, medication use, fluid restrictions, and diet recommendations in HD patients. Furthermore, it was found that the intervention program and the telephone follow-up made an improvement in the mean scores of laboratory values, i.e., serum potassium, calcium, creatinine, phosphate, albumin, iron, BUN, hemoglobin, nPCR, and Kt/V. Therefore, we need to make changes in terms of patient education strategies and utilize effective methods in this regard in ESRD patients. Overall, there are different factors affecting the adherence to the therapeutic regimen in patients undergoing HD. In this regard, factors such as socioeconomic status, health beliefs, patients’ attitudes towards treatment, and cultural differences are notable. Adherence to dietary recommendations, fluid restrictions, and medication regimen are not easy. On the other hand, neglecting the therapeutic regimen may lead to serious consequences, so that low patient education level can cause the patients to lack knowledge about the disease process and related therapeutic regimen.

In line with the results of our study, Kamrani et al. (2015) showed that patient education and nurse-led telephone follow-up (tele-nursing) could improve treatment adherence in patients with acute coronary syndrome [30]. Alikari et al. (2015) used counseling intervention and active participation in clinical decision-making to improve of treatment adherence. They revealed that patients’ active involvement in the educational program improved their awareness and perception, which led to a higher level of treatment adherence in HD patients [31]. The improvement in treatment adherence results in a better physical condition and treatment process in HD patients. Based on the results of a study by Durose et al. (2004), it was found that the use of patient education techniques can motivate HD patients to comply with dietary restrictions, which in return can lead to weight loss in these patients. Patient education about dietary restrictions can reduce the risk of physical complications, improve patients’ quality of life, and increase life expectancy by 20 years or more [32]. Some studies have shown that educational intervention increases patients’ knowledge and alters patients’ attitudes towards the disease, through which patients can adopt a better cooperative attitude towards adherence to the therapeutic regimen [33]. The nurse-led telephone follow-up not only improved the efficiency of patient education but also increased the duration of adherence to the therapeutic regimen. Since the patients easily forget the medical advice, repetition of the orders and key points, and the nurse-led telephone follow-up enable them to better memorize the therapeutic regimen. Nurses can examine the patient’s needs and then refer him/her to a health professional, if necessary. Therefore, care services can be provided based on the patients’ needs [24].

In line with our results, the previous studies have reported that there was a moderate adherence level for fluid and dietary restrictions in HD patients [34, 35], which indicates the educational need of HD patients. Therefore, nurses can play an important role in improving HD adherence by providing training in new methods, especially tele-nursing. Kreps et al. (2011) Showed that motivational messages increased medication adherence in patients with chronic diseases [36], which is consistent with the results of the present study. Sending a message by the nurse to remind patients of their daily medication intake is important and thus improves adherence to the medication regimen. Karam et al. (2017) showed that there was no statistically significant relationship between treatment adherence and serum phosphorus in patients with HD [37]. Our study also showed that there was no statistical relationship between patient education and nurse-led telephone follow-up (tele-nursing) and serum sodium level. This indicates that the ESRD-AQ may not be an appropriate tool to assess the relationship between treatment adherence and more biochemical indexes in patients with HD.

Previous studies have indicated that the patient education program with telephone follow-up has positive effects on patients with chronic conditions so that it improves and alters health behavior and promotes cooperative attitudes in these groups of the patients. Furthermore, patients who received nurse-led telephone follow-up showed better adherence to therapeutic regimens and had more physical activity and notable changes in their behavior compared to those who only received routine care [38, 39]. The results of the above studies are consistent with the results of our study.

Beaver et al. (2009), in a study on patients with breast cancer, concluded that the ineffectiveness of telephone follow-up might be due to the effects of the disease on patient’s ability to accept his/her condition [40]. Hung et al. (2014) conducted a study on the impact of telephone-delivered nutrition and exercise counseling on nutritional status, body composition, and quality of life in patients undergoing peripheral blood stem cell transplantation. Based on the results of their study, it was indicated that telephone-delivered counseling alone might not decrease hospital readmission and there is a need for other intervention methods of patient education [41]. The results of the studies by Beaver et al. (2009) and Hung et al. (2014) are inconsistent with the results of our study.

In addition to the nurse-led telephone follow-up, we applied patient education booklets to provide further education in this study. It is recommended to provide a complete education for patients using educational pamphlets, booklets, videos, and other tools before follow-up. All the points that the patient needs to know, including disease progress, complications, treatment process, medication, and diet, should be educated. The patient should acquire in-depth knowledge on his/her condition. In this regard, telephone follow-up and home visits are recommended to be used along with patient education. This study highlights the importance of patient education with nurse-led follow-up program in patients with chronic diseases, especially HD patients. Some patients have poor treatment adherence due to the lack of knowledge and perception of the disease. Accordingly, the patient education program and nurse-led follow-up are recommended to improve patients’ perception and knowledge on their chronic conditions. Moreover, frequent follow-ups can increase treatment adherence and provoke patients to follow the medical advice at home, although the necessity of follow-up decreases as patients gain more knowledge on their condition. Besides, the patients should not be left on their own, and regular follow-ups should be a part of home care as even well-informed patients have periods of neglecting medical advice when they are on long-term medication for their condition [31].

One of the limitations of this study was the short-term follow-up period due to grant limitations and large groups of the study. Another limitation was the small sample size that could affect the results because there was a dialysis center located throughout Urmia city. Therefore, it is suggested that future studies in this field be done with larger sample size. The use of a convenience sampling method in this study was also a major limitation because it is associated with a significant risk of selection bias. It is recommended that future studies include other sampling methods. Since this study was not a multi-center trial, leading to low generalizability of the study results. Moreover, some patients failed to attend the HD sessions as scheduled due to financial difficulties, and this issue could affect treatment adherence in these patients. Differences in mental and spiritual characteristics, motivations, and the personality traits of the participants might have affected their perception and knowledge and their treatment adherence. The above limitations were beyond the researchers’ control. Another limitation was that some patients did not answer the telephone in the first call, so that the researchers tried to call them again, which made the intervention time-consuming.

## Conclusions

We concluded that the patient education program and nurse-led telephone follow-up could improve HD adherence and modify health behavior in patients with ESRD by increasing their knowledge on their chronic conditions. The mean score of overall treatment adherence in the intervention group increased to the highest level at the final assessment. This indicates that continuous follow-up improves treatment adherence in ESRD patients. Therefore, the results support the necessity of continuous care and nurse-led follow-up for HD patients.

## Supplementary Information

**Additional file 1.** The list of items related to the four dimensions of ESRD-AQ including hemodialysis attendance, medication use, fluid restrictions, and diet recommendation.

**Additional file 2.** CONSORT 2010 checklist of information to include when reporting a randomised trial*.

## Data Availability

The datasets used and/or analysed during the current study are available from the corresponding author on reasonable request.

## Abbreviations

ESKD: End-Stage Kidney Disease
CKD: Chronic Kidney Disease
HD: Hemodialysis
ESRDAQ: End-Stage Renal Disease Adherence Questionnaire
eGFR: Estimated Glomerular Filtration Rate
PD: Peritoneal Dialysis
RRT: Renal Replacement Therapy
BUN: Blood Urea Nitrogen
nPCR: The normalized Protein Catabolic Rate
