# Supplementary material for: Do the patient education program and nurse-led telephone follow-up improve treatment adherence in hemodialysis patients? A randomized controlled trial
Source: BMC Nephrol. 2021 Apr 7;22:119. doi: 10.1186/s12882-021-02319-9 (PMC8028152; doi:10.1186/s12882-021-02319-9)
Supplement: Supplementary file 1 — Additional file 1. The list of items related to the four dimensions of ESRD-AQ including hemodialysis attendance, medication use, fluid restrictions, and diet recommendation. [file 12882_2021_2319_MOESM1_ESM.docx]

| **Additional file 1:** The list of items related to **the four** dimensions of ESRD-AQ including hemodialysis attendance, medication use, fluid restrictions, and diet recommendation**.** | |
| --- | --- |
| **Items** | **Diet recommendations** |
| - This week(1) - Last week(2) - One month ago(3) - More than a month ago(4) - When I first began dialysis treatment(5) - Never(6) - Other (Specify)(7): ____________ | When was last time a medical professional (your doctor, nurse, dietician, or other medical staff) talked to you about your diet? |
| - Every dialysis treatment(1) - Every week(2) - Every month(3) - Every 2 to 3 months(4) - Every 4 to 6 months(5) - When I have abnormal blood or other (for example, blood pressure) test results(6) - Rarely(7) - Irregularly(8) - Never(9) - Other (Specify)(10): ____________ | How often does a medical professional (your doctor, nurse, dietician or other medical staff) talk to you about the importance of following a proper diet? |
| - Highly important(1) - Very important(2) - Moderately important(3) - A little important(4) - Not important(5) | How important do you think it is to watch the types of food you eat each day? |
| - Because I fully understand that my kidney condition requires to watch my diet(1) - Because watching my diet is important to keep my body healthy(2) - Because a medical professional (my doctor, nurse, or dietician) told me to do so(3) - Because I got sick after eating certain food that I was not supposed to eat(4) - Because I was hospitalized after eating certain food that I was not supposed to eat(5) - I don't think watching my diet is important to me(6) - Other (Specify)(7): ____________ | Why do you think it is important for you to watch your diet daily? (Please choose one best answer that applies to you.) |
| - No(1) - Yes(2) | Have you had any difficulty following your dietary recommendations? |
| - No difficulty(1) - A little difficulty(2) - Moderate difficulty(3) - A lot of difficulty(4) - I was unable to follow any recommendations at all(5) | How much difficulty have you had following your dietary recommendations? |
| - Not applicable: No difficulty(1) - I was not willing to control what I want to eat(2) - I was unable to avoid certain un recommended food(3) - I don't understand what type of diet to follow(4) - Other (Specify)(5): ____________ | What type of difficulty have you had keeping your dietary recommendations? |
| - All of the time(1) - Most of the time(2) - About half of the time(3) - Very seldom(4) - None of the time(5) | During the past week, how many times have you followed the diet recommendations? |
| **Items** | **Fluid restrictions** |
| - This week(1) - Last week(2) - One month ago(3) - More than a month ago(4) - When I began dialysis treatment(5) - Never(6) - Other (Specify)(7): _____________ | When was the last time a medical professional (your doctor, nurse or dietician or other medical staff) spoke to you about your fluid restrictions? |
| - Every dialysis treatment(1) - Every week(2) - Every month(3) - Every 2 to 3 months(4) - Every 4 to 6 months(5) - When I have abnormal blood or other (for example, blood pressure) test results(6) - Rarely(7) - Irregularly(8) - Never(9) - Other (Specify)(10): _____________ | How often does a medical professional (your doctor, nurse, dietician or other medical staff) talk to you about the importance of fluid restriction? |
| - All of the time(1) - Most of the time(2) - About half of the time(3) - Very seldom(4) - None of the time(5) | During the past week, how often have you followed the fluid restriction recommendations? |
| - Highly important(1) - Very important(2) - Moderately important(3) - A little important(4) - Not important(5) | How important do you think it is to limit your fluid intake? |
| - Because I fully understand that my kidney condition requires limiting fluid intake(1) - Because limiting fluid intake is important to keep my body healthy(2) - Because a medical professional (my doctor, nurse, dietician, or other medical staff) told me to do so(3) - Because I got sick after I drank lots of fluid(4) - Because I was hospitalized after I drank lots of fluid(5) - I don't think limiting fluid is very important to me(6) - Other (Specify)(7): _____________ | Why do you think it is important for you to limit your fluid intake? (Please choose one best answer that applies to you.) |
| - No(1) - Yes(2) | Have you had any difficulty with limiting your fluid intake? |
| - No difficulty(1) - A little difficulty(2) - Moderate difficulty(3) - A lot of difficulty(4) | How much difficulty have you had following your fluid restriction recommendations? |
| - No difficulty(1) - Not interested(2) - I was unable to control fluid intake(3) - I don't understand how to follow the fluid restriction(4) - Other(5): ____________ | If you had difficulty following your fluid restriction recommendations, what type of difficulty have you had? |
| - More than 3 times(1) - 3 times(2) - Twice(3) - Once(4) - None of the time(5) - Other(6): ____________ | During the past week, how many times have you weighed yourself at home (outside dialysis center)? |
| - Highly important(1) - Very important(2) - Moderately important(3) - A little important(4) - Not important(5) | How important do you think it is to weigh yourself daily? |
| **Items** | **Medication use** |
| - This week(1) - Last week(2) - One month ago(3) - More than a month ago(4) - When I first began dialysis treatment(5) - Never(6) - Other (Specify)(7): ____________ | When was the last time a medical professional (your doctor, nurse, dietician or other medical staff) spoke to you about your medicines? |
| - Every dialysis treatment(1) - Every week(2) - Every month(3) - Every 2 to 3 months(4) - Every 4 to 6 months(5) - When I have abnormal blood or other (for example, blood pressure) test results(6) - Rarely(7) - Irregularly(8) - Never(9) - Other (Specify)(10): ____________ | How often does a medical professional (your doctor, nurse, dietician or other medical staff) talk to you about the importance of taking medicines as ordered? |
| - Highly important(1) - Very important(2) - Moderately important(3) - A little important(4) - Not important(5) | How important do you think it is to take your medicines as scheduled? |
| - Because I fully understand that my kidney condition requires to take medicines as scheduled(1) - Because taking medicines is important to keep my body healthy(2) - Because a medical professional (my doctor, nurse, dietician, or other medical staff) told me to do so(3) - Because I had an experience that I was sick after I missed medicines(4) - Because I had an experience that I was hospitalized after I missed medicines(5) - I don't think taking medicines is very important to me(6) - Other (Specify)(7): ____________ | Why do you think it is important to take your medicines as scheduled? (Please choose one best answer that applies to you.) |
| - No(1) - Yes(2) | Have you had any difficulty with taking your medicines? |
| - No difficulty(1) - A little difficulty(2) - Moderate difficulty(3) - A lot of difficulty(4) - Extreme difficulty(5) | How much difficulty have you had with taking your prescribed medicines? |
| - None of the time: I did not miss my medicines(1) - Very seldom(2) - About half of the time(3) - Most of the time(4) - All of the time(5) | During the past week, how often have you missed your prescribed medicines? |
| - Not applicable: I did not miss medicines(1) - Forgot to take medicines(2) - Forgot to order medicines(3) - Medicine cost(4) - Inconvenience(5) - I was hospitalized(6) - Side effects(7) - Other(8): _____________ | What was the main reason for not taking your prescribed medicines this past week? |
| - Loss of appetite(1) - Nausea/vomiting/diarrhea/constipation(2) - Stomach pain(3) - Dizziness(4) - Headache(5) - Itching/skin problems(6) - Other (Specify symptoms)(7): _____________ | (Answer this question when you have marked the above question as “Side effects.”) What kind of side effect(s) to the medication(s) did you have? (Please choose one best answer that applies to you.) |
| **Items** | **Hemodialysis attendance** |
| - 2 days or less(1) - 3 days(2) - 4 days(3) - More than 4 days(4) - More than 5 days(5) | How many days a week do you receive hemodialysis treatment? |
| - Less than 3 hours(1) - 3 hours(2) - 3 hours and 15 minutes(3) - 3 hours and 30 minutes(4) - 3 hours and 45 minutes(5) - 4 hours(6) - More than 4 hours(7) - Other (Specify the hours)(8): ____________ | How many hours are you treated for each hemodialysis? |
| - Yes(1) - No, because I have to come to the dialysis center too early(2) - No, because I have to come to the dialysis center too late(3) - No, because of my work schedule(4) - No, because it is my meal time and I get hungry during dialysis treatment(5) - No, because it is my medication time and I have to take medicines/insulin(6) - No, because of (Other)(7): ____________ | Is your dialysis schedule convenient for you? (Please choose one best answer that applies to you.) |
| - This week(1) - Last week(2) - One month ago(3) - More than a month ago(4) - When I first began dialysis treatment(5) - Never(6) - Other (Specify)(7): ___________ | When was the last time a medical professional (your doctor, nurse, dietician, or other medical staff) talked to you about the importance of not missing your dialysis treatment? |
| - Every dialysis treatment(1) - Every week(2) - Every month(3) - Every 2 to 3 months(4) - Every 4 to 6 months(5) - When I have abnormal blood or other test results(6) - Rarely(7) - Irregularly(8) - Never(9) - Other (Specify)(10): ___________ | How often does a medical professional (your doctor, nurse, dietician, or other medical staff) talk to you about the importance of staying for the entire dialysis time during your dialysis treatment? |
| - Highly important(1) - Very important(2) - Moderately important(3) - A little important(4) - Not important(5) | How important do you think it is to follow your dialysis schedule? |
| - Because I fully understand that my kidney condition requires dialysis as scheduled(1) - Because following the dialysis schedule is important to keep my body healthy(2) - Because medical professional (my doctor, nurse, or dietitian) told me to do so(3) - Because I had an experience that I was sick after I missed dialysis(4) - Because I had an experience that I was hospitalized after I missed dialysis(5) - I don't think following the dialysis schedule is very important to me(6) - Other (Specify)(7): ____________ | Why do you think it is important to follow your dialysis schedule? (Please choose one best answer that applies to you.) |
| - No difficulty(1) - A little difficulty(2) - Moderate difficulty(3) - A lot of difficulty(4) - Extreme difficulty(5) | How much difficulty have you had staying for your entire dialysis treatment as ordered by your doctor? |
| - None (I did not miss any treatments)(1) - Missed one dialysis treatment(2) - Missed two dialysis treatments(3) - Missed three dialysis treatments(4) - Missed four or more dialysis treatments(5) | During the last month, how many dialysis treatments did you miss completely? |
| - Not applicable: I did not miss any treatment(1) - Transportation problems(2) - I had other things to do (Please explain)(3): - Hemodialysis access (graft, fistula, or catheter) clotted(4) - Physician (medical or surgical) appointment(5) - I had to go to the emergency room(6) - I was hospitalized(7) - Forgot(8) - “Didn't want to go” or “Couldn't go”(9) - Other (Please specify)(10): ____________ | What was the main reason you missed your dialysis treatment last month? |
| - Because dialysis treatment makes me anxious(1) - Because I had vomiting/diarrhea(2) - Because I had cramping(3) - Because I often get hungry during dialysis treatment(4) - Because I was physically uncomfortable (Specify the condition)(5) - Because I was sick due to other conditions (Specify the conditions)(6) - Because I was emotionally depressed(7) - Other(8): ____________ | (Answer this question when you marked the above question as “Didn't want to go Couldn't go.”) Why didn't you want to go to the dialysis center? (Please choose one best answer that applies to you) |
| - Not applicable: I have not shortened my dialysis time(1) - Once(2) - Twice(3) - Three times(4) - Four to five times(5) - Other (Specify frequency)(6): ____________ | During the last month, how many times have you shortened your dialysis time? |
| - Not applicable: I have not shortened my dialysis time(1) - Less than 10 minutes or 10 minutes(2) - 11 to 20 minutes(3) - 21 to 30 minutes(4) - More than 31 minutes(5) - Other (Specify)(6) (If you need to write two or more different time because you shortened dialysis more than once, please use this space): _____________________________ | During the last month, when your dialysis treatment was shortened, what was the average number of minutes? |
| - Not applicable: I have not shortened my dialysis time(1) - Cramping(2) - Bathroom use(3) - Restlessness(4) - Low blood pressure(5) - Access (graft, fistula, or catheter) clotted(6) - Physician (medical or surgical) appointment(7) - Personal business or emergency(8) - Work schedule(9) - Transportation problems(10) | What was the main reason you have shortened your dialysis treatment? |
